# *Haemophilus influenzae* Carriage among Healthy Children in Portugal, 2015–2019

**DOI:** 10.3390/microorganisms10101964

**Published:** 2022-10-04

**Authors:** Maria Paula Bajanca-Lavado, Luís Cavaco, Mariana Fernandes, Tiago Touret, Catarina Candeias, Alexandra S. Simões, Raquel Sá-Leão

**Affiliations:** 1 *Haemophilus influenzae* Reference Laboratory, Department of Infectious Diseases, National Institute of Health, 1600-046 Lisboa, Portugal; 2 Laboratory of Molecular Microbiology of Human Pathogens, Instituto de Tecnologia Química e Biológica António Xavier, Universidade Nova de Lisboa, 2780-157 Oeiras, Portugal

**Keywords:** *Haemophilus influenzae*, serotype, MLST, carriage, children, surveillance, antimicrobial resistance, capsule, whole genome sequencing, day-care center

## Abstract

*Haemophilus influenzae* is an important cause of mucosal and invasive infections and a common colonizer of the upper respiratory tract. As there are no recent data on *H. influenzae* carriage in Portugal, we aimed to characterize carriage samples and investigate possible parallelisms with disease isolates. Between 2016–2019, 1524 nasopharyngeal samples were obtained from children (0–6 years) attending day-care. *H. influenzae* were serotyped and screened for β-lactamase production. Strains producing β-lactamase and/or those that were encapsulated were further characterized by antibiotype; encapsulated strains were also investigated for MLST and the presence of antimicrobial resistance and virulence genes (extracted from whole genome sequencing). The overall carriage rate was 84.1%. Most isolates (96.7%) were nonencapsulated. Encapsulated strains were of serotypes f (1.8%), e (1.1%), a (0.3%), and b (0.1%). MLST showed clonality within serotypes. Although the lineages were the same as those that were described among disease isolates, colonization isolates had fewer virulence determinants. Overall, 7.5% of the isolates were β-lactamase positive; one isolate had *bla_TEM-82_*, which has not been previously described in *H. influenzae*. A single isolate, which was identified as *H. parainfluenzae*, had an incomplete f-like cap locus. In conclusion, circulation of serotype b is residual. The few encapsulated strains are genetically related to disease-causing isolates. Thus, surveillance of *H. influenzae* carriage should be maintained.

## 1. Introduction

*Haemophilus influenzae* is a Gram-negative bacterium that colonizes the human upper respiratory tract (URT), where it can remain asymptomatic [1,2,3]. It can also progress from colonizer to pathogen and cause mucosal or invasive infections, particularly in children [4,5,6,7]. *H. influenzae* is classified based on the production and antigenicity of a polysaccharide capsule. Strains that produce capsule are designated as encapsulated and are divided into six serotypes (Hia to Hif) [8]; nonencapsulated strains are designated as non-typable (NTHi). The capsular operon, or “cap locus”, is composed of three unique functionally distinct regions, which are designated I, II, and III. This organization is the same for all encapsulated strains, with region II being serotype-specific [9,10,11].

Before the implementation of highly effective Hib conjugate vaccines in the immunization programs of most countries worldwide, strains of *H. influenzae* of serotype b (Hib) were the leading cause of bacterial meningitis in children under five years of age [12,13,14,15,16]. Upon implementation of this vaccine, the epidemiology of invasive *H. influenzae* disease changed dramatically with a pronounced decline in Hib incidence [17,18,19,20,21,22,23,24]. Nowadays, with the widespread use of Hib vaccine, NTHi has become the main cause of *H. influenzae* invasive disease [25,26,27,28,29]. In addition, some non-b serotypes—Hie and Hif, in particular—have also been increasingly identified in invasive disease [18,19,20].

In Portugal, the Hib conjugate vaccine was introduced in the National Immunization Plan (NIP) in 2000, with a schedule of three doses, plus a booster dose, to be administered at 2, 4, 6, and 18 months of age [30]. The epidemiology of *H. influenzae* invasive disease has been studied in Portugal before and after the implementation of the Hib vaccine in the NIP [27,31,32]. These studies demonstrated that there was a pronounced decline in the relative proportion of invasive disease cases due to Hib, from 81.0% in 1989–2001, to 13.2% and 13.5% in 2002–2010 and 2011–2018, respectively, while the NTHi increased from 19.0% to 77.1% to 79.2% in the same periods. Non-b serotypes have been reported since 2001, with the characterization of the first Hif isolate [31]. More recently, 3.1% of non-b invasive disease isolates were identified as Hif, 2.7% were identified as Hia, and 1.5% were identified as Hie [27].

*H. influenzae* colonization has also been studied in Portugal, albeit sporadically, since 1996 in children [33,34,35,36]. In the first study, which was performed in 1996 with 586 healthy children aged 6–72 months and attending day-care centers in Lisbon, 72% of the children carried *H. influenzae* in the nasopharynx and 3.8% of the isolates were Hib [35]. The second study was a longitudinal study that followed, for one year (February 1998-February 1999), 47 children 14–37 months of age who attended a single day-care center in Lisbon. Among 414 nasopharyngeal samples, *H. influenzae* was isolated in 87% of the samples [34]. The third study, performed in Coimbra in February of 2009, enrolled 585 children 4–74 months of age, attending day-care; the *H. influenzae* carriage rate was 33% [33]. Finally, between 1997 and 2000, carriage was evaluated among children (≤12 years of age) attending a Pediatric Emergency Department of a Hospital in Lisbon; among the 466 children that were enrolled, 41% were nasopharyngeal carriers of *H. influenzae* [36].

Studies conducted in other countries on *H. influenzae* colonization among children attending day-care suggest that colonization varies among settings, with colonization rates of 27% described in Spain [37], 37% in the Netherlands [38], 41% in France [39], and 72% in Brazil [40], for example. 

*H. influenzae* infections are generally treated with β-lactams antibiotics. Isolates resistant to this class of antibiotics have been described since the early 1970s. Two different β-lactam resistance mechanisms are well known: one is the enzymatic hydrolysis of β-lactams by TEM-1 or ROB-1 β-lactamases [41,42,43], the other is a decrease in β-lactam affinity for altered penicillin-binding protein 3 (PBP3) due to mutations in the *ftsI* gene [44,45]. Isolates with this latter mechanism are called β-lactamase-negative ampicillin-resistant (BLNAR), while isolates with both mechanisms are named β-lactamase-positive amoxicillin/clavulanic acid-resistant (BLPACR) for their decreased susceptibility, or resistance, to amoxicillin/clavulanic acid [46,47].

Among Portuguese invasive disease isolates, the proportion of β-lactamase producing strains declined overtime from 26.9% in pre-vaccine era [31] to 10.4% and 13.5%, respectively, in the two post-vaccine studies [27,32]. Similarly, among Portuguese colonizing isolates, the proportion of β-lactamase-producing strains also declined over time from 20% in pre-vaccine era [35] to 12% in the post-vaccine period [34]. 

In recent years, the National Reference Laboratory has identified an increasing number, albeit small, of Hib cases due to vaccine failure [27]. Whether a similar surge of Hib strains occurred in colonization was unknown. This fact, associated with the lack of recent data regarding the epidemiology of *H. influenzae* colonization among Portuguese children, led us to conduct the current study. 

Here, we aimed to characterize, phenotypically and genotypically, *H. influenzae* strains circulating in Portugal among asymptomatic children attending day-care centers from 2015 to 2019.

## 2. Materials and Methods

### 2.1. Ethics Statement

The study was registered and approved by the Ethics Research Committee of the NOVA Medical School/Faculdade de Ciências Médicas—Universidade Nova de Lisboa (CEFCM) (30/2017/CEFCM). The directors of all participating day-care centers approved the study. Written signed informed consent was obtained from parents or guardians of participating children. All samples and questionnaires were numerically coded at the time of sample collection and processed anonymously thereafter. Data were stored in a dedicated secure database. 

### 2.2. Study Design

Between January and March of 2015, 2016, 2018 and 2019, cross-sectional prevalence colonization studies were conducted. Nasopharyngeal samples from healthy children attending day-care centers in the Oeiras region were obtained from children up to 6 years of age. The day-care centers served populations of different socio-economic strata.

### 2.3. Questionnaires

Information regarding children’s age, gender, antimicrobial use (at sampling, in the previous month and in the previous six months) and recent illnesses and hospitalizations was obtained through questionnaires filled in by the parents or guardians.

### 2.4. Sample Collection

Nasopharyngeal swabs were collected by a trained pediatric nurse as previously described [48]. Briefly, a neonatal FLOQSwabs^TM^ (Copan Flock Technologies, Brescia, Italy) nylon flocked swab was inserted through one nostril until the nasopharynx was reached. The swabs were then immediately introduced into a tube containing 1 mL of skim milk tryptone-glucose-glycerol (STGG). During transportation to the laboratory, tubes were placed on wet ice. Upon arrival to the laboratory, samples were vortexed for 20 s and frozen at −80 °C [49]. 

### 2.5. Isolation of Putative Haemophilus influenzae

Samples were plated into selective chocolate agar plate containing Isovitalex and bacitracin (Becton, Dickinson and Company, Sparks, NV, USA), and incubated overnight at 37 °C in 5% CO_2_-enriched atmosphere. Presumptive identification of *H. influenzae* was made based on growth in the selective medium and colony morphology. Suspected colonies were picked and streaked into chocolate agar plates containing Isovitalex. Pure cultures were frozen at −80 °C in Mueller–Hinton broth containing 15% (*v*/*v*) glycerol.

### 2.6. Preparation of DNA Templates for Serotyping

Four to six colonies of an overnight culture were resuspended in 100 μL of sterile water and boiled for 15 min. After centrifugation at 13,000 rpm at room temperature, 90 μL of the supernatant (containing DNA) was removed and used as template.

### 2.7. Serotyping

The presence or absence of the polysaccharide capsule was screened for by a polymerase chain reaction (PCR) of *bexA* gene (region I of the cap locus and energy-coupling component of the capsule export apparatus). Capsular type was determined by amplification of capsule-specific genes (for serotypes a–f) using primers and conditions previously described [50].

### 2.8. β-Lactamase Production and Minimum Inhibitory Concentration (MIC) Determination

β-lactamase production was investigated for all isolates by the chromogenic cephalosporin assay using nitrocefin as subtract (Oxoid Limited, Hampshire, UK). ATCC^®^10211 (BLNAS, β-lactamase-negative ampicillin-susceptible), ATCC^®^49247 (BLNAR, β-lactamase-negative ampicillin-resistant) and NCTC^®^11315 (BLPAR, β-lactamase-positive ampicillin-resistant) were used as control strains for susceptibility testing.

Antibiotic susceptibility testing was performed for encapsulated isolates and/or β-lactamase producing strains using the broth microdilution method using commercially available MIC panels (MICroSTREP plus^®^, Beckman Coulter, Pasadena, CA, USA) following the latest European Committee on Antimicrobial Susceptibility Testing (EUCAST) guidelines and breakpoints established for *H. influenzae* [51]. The antimicrobial agents tested were: ampicillin, amoxicillin/clavulanic acid, cefotaxime, cefuroxime, cefepime, meropenem, ciprofloxacin, chloramphenicol, tetracycline, rifampin, and trimethoprim-sulfamethoxazole.

### 2.9. DNA Extraction and Whole Genome Sequencing (WGS)

DNA extraction and WGS were performed for the following selected isolates: all capsulated strains, one NTHi strain with an uncommon resistance phenotype (BLPACR phenotype, being the only beta-lactamase producer isolate that was resistant to amoxicillin-clavulanate, a rare phenotype among Portuguese isolates), and one strain of *Haemophilus parainfluenzae* that, by PCR, gave a positive result for the capsular gene *bexA*.

Total DNA was extracted using the MagNA Pure Compact (Roche, Basel, Switzerland) automated system. Briefly, strains were grown overnight at 37 °C with 5% CO_2_ in chocolate agar plates supplemented with Isovitalex. For each pure culture, half of the culture plate was suspended into 1 mL of PBS solution; 200 μL were transferred into a sample tube containing 200 μL of lysis buffer (Roche) and 1.74 mg of RNase A (Merck, Kenilworth, NJ, USA) and incubated for 20 min at 37 °C. The total DNA of each sample was extracted using the MagNA Pure Compact System (Roche) following the manufacturer’s instructions.

DNA quality was assessed through determination of A260/A280 and A260/A230 ratios using Nanodrop. DNA quantification was performed using the dsDNA High Sensitivity Qubit kit (ThermoFisher, Waltham, MA, USA), according to the manufacturer’s instructions.

WGS was performed at the Genomics Unit of Instituto Gulbenkian de Ciências, Oeiras. Isolates were sequenced using the Illumina NextSeq 500 platform (Illumina, San Diego, CA, USA) to an expected coverage of 100-fold using pair-ended reads with 150 bp per read.

### 2.10. Whole Genome Sequencing Analysis

Assembly of the genomic sequences, quality control and quality assurance were performed using INNUca v4.2.2 (https://github.com/B-UMMI/INNUca last accessed on 25 October 2020) under default parameters. Genome size, number of contigs and multilocus sequence typing (MLST) were determined using INNUca. hicap v1.0.3 [9] was used to determine and confirm, *in silico*, the capsular serotype of encapsulated isolates.

Genome annotation was made using Prokka v1.13.3 [52], with a custom database consisting of the protein sequences of all 69 complete *H. influenzae* genomes present until now in NCBI (accessed on 30 June 2020).

To determine the presence/absence of virulence and resistance genes in the genomes, a BLAST-based analysis using ABRicate v1.0.1 (https://github.com/tseemann/ABRicate last accessed on 25 October 2020) was performed with an identity and coverage cutoff of 80% for each of them. For the detection of virulence genes, a custom database, created with all 105 *H. influenzae* virulence genes described in Virulence Factor Database [53] and by Wong and colleagues [54], was used. The only exception to this approach was that, for the *iga1* gene (which is very variable, and so, the use of a single sequence might lead to false negative results), we performed BLAST using the gene in the reference strain (Rd KW20, accession code: NC_000907) to extract a sequence for an isolate of each serotype; these sequences were then used in ABRicate for the detection of the *iga1* gene in all other isolates. Detection of resistance genes was performed using the CARD database (https://card.mcmaster.ca/, accessed on 29 June 2020) [55]. Detection of altered PBP3 alleles was also performed with this software using a database available at PubMLST (https://pubmlst.org/bigsdb?db=pubmlst_hinfluenzae_seqdef&page=locusInfo&locus=ftsI, accessed on 26 June 2020).

### 2.11. Accession Numbers

Read data and assembled and annotated contigs of the 43 sequenced isolates were deposited in the NCBI database under BioProject accession number PRJNA824278.

### 2.12. Statistical Analysis

The chi-square and *t*-student tests were used when appropriate to compare demographic and epidemiological data regarding the population under study in each year. The proportion of colonization with *H. influenzae* by child’s age group and per year was compared. For that, standard errors for colonization rates per age group were calculated and a chi-square test was used to compare them.

## 3. Results

### 3.1. Population Characteristics

The characteristics of the population under study are summarized in Table 1. A total of 1524 nasopharyngeal samples were obtained from healthy children: 315 in 2015, 318 in 2016, 427 in 2018, and 464 in 2019. The children that participated in the study had a mean age of 3 years (39.3 months), and 53.6% of them were male. Data on antibiotic use were known, in at least one category, for 1491 of the children: 4.3% were taking antibiotics at the time of sample collection, 17.6% in the previous month and 36.1% in the previous 6 months.

### 3.2. Colonization Rates and Capsular Type

Presumptive *H. influenzae* were isolated according to growth characteristics in selective medium and morphology of colonies. The overall carriage rate was 84.1%: children that were 13–24-months-old were the most colonized (92.1%), while the oldest ones (over 60 months) were the least colonized (75.3%) (Table 2).

Capsular genotyping by PCR showed that most carriers (96.7%) were colonized by nonencapsulated *H. influenzae* (NTHi) (1240/1282). Encapsulated strains were of serotypes f (23/1282; 1.8%), e (14/1282; 1.1%), a (4/1282; 0.3%) and b (1/1282; 0.1%) (Table 2). Encapsulated strains were found in all sampling periods. One additional isolate, which was further identified as *H. parainfluenzae,* had an amplification product for the *bexA* gene, and a non-specific amplification was obtained when using primers for *capD*. This isolate was further characterized by whole genome sequencing (see details below).

### 3.3. β-Lactamase Production and Antimicrobial Susceptibility Testing

β-lactamase production test was evaluated for all putative 1282 *H. influenzae* isolates. Overall, 7.5% of them were β-lactamase producers (96/1282). This value was higher among encapsulated isolates (11.9%; 5/42; three isolates of serotype e and two isolates of serotype f) than among NTHi isolates (7.3%; 91/1240), albeit this difference was not statistically significant (*p* = 0.27). The proportion of isolates that were β-lactamase producers decreased significantly in 2019 (9.3% in 2015, 9.5% in 2016, 8.5% in 2018 and 4.0% in 2019; *p* = 0.001).

Antimicrobial susceptibility was determined for 133 isolates: all β-lactamase producing isolates (*n* = 96) and all encapsulated isolates (*n* = 42; this includes five β-lactamase positive isolates) (Table 3).

All β-lactamase-producing isolates were resistant to ampicillin and had an MIC ≥ 4 mg/L. One β-lactamase producer isolate was also resistant to amoxicillin-clavulanic acid (MIC = 4 mg/mL), and therefore, it was phenotypically classified as BLPACR (beta-lactamase-positive amoxicillin/clavulanate-resistant). Of note, one NTHi isolate was phenotypically resistant to ciprofloxacin (MIC = 0.12 mg/L). Three NTHi isolates were multidrug resistant (i.e., they were resistant to three or more antimicrobial classes).

All β-lactamase-producing isolates were susceptible to cefotaxime, meropenem and rifampicin, and non-susceptible to cefuroxime (90.6% showed intermediate resistance (0.25 ≤ MIC < 2 mg/L), and 9.4% were resistant (MIC ≥ 2 mg/L)). In addition, more than one third (34.4%) of the isolates were resistant to trimethoprim-sulfamethoxazole (MIC ≥ 2 mg/L) (Table 3).

Among the encapsulated isolates, five were resistant to ampicillin (MIC = 8 mg/L). These isolates were reported above as beta-lactamase producers. All encapsulated isolates were susceptible to amoxicillin-clavulanic acid, cefotaxime, ciprofloxacin, chloramphenicol, meropenem, tetracycline, and rifampicin. All encapsulated isolates were classified as intermediately resistant to cefuroxime. One isolate was resistant to cefepime, and two isolates were resistant to trimethoprim-sulfamethoxazole (Table 3).

### 3.4. Whole Genome Sequencing (WGS)

Forty-three samples were analyzed by WGS: 41 encapsulated strains (all encapsulated strains with the exception of one serotype f strain isolated in 2019 that was lost during the study), one NTHi strain that was resistant to ampicillin and amoxicillin-clavulanate, and one strain of *Haemophilus parainfluenzae* that, by PCR, appeared to have capsular genes. Upon genome assembly, genome coverage ranged between 25× and 352×; the number of contigs per strain ranged from 20 to 101. Genome size ranged between 1.76 and 1.90 Mbp (Table 4). The genome size of the *H. parainfluenzae* strain (PT11474) was 2.0 Mbp.

### 3.5. Multilocus Sequence Typing (MLST)

The sequence type (ST) of all sequenced *H. influenzae* strains (*n* = 42) was determined (Table 4). Seven STs were identified among the encapsulated strains. All serotype a samples were of ST23, whilst the serotype b sample was of ST6. Samples of serotypes e were separated into two STs (ST18 and ST122, with seven isolates each), and samples of serotype f were separated into three STs: ST124 (*n* = 16), ST973 (*n* = 5) and ST2346 (*n* = 1). ST2346 is firstly described in this study. The STs within serotypes e and f were single locus variants, indicating that they were related to each other. The NTHi resistant isolate was of ST145.

### 3.6. Genetic Determinants of Antimicrobial Resistance

We investigated resistance genes and mutations in genes that are associated with antimicrobial resistance in all sequenced strains and compared the results with those obtained by antimicrobial susceptibility testing.

The *bla* gene was detected in all β-lactamase positive samples: *bla*_TEM-1_ was identified in samples PT10981, PT11022, PT11604 and PT12019 whereas *bla*_TEM-82_ was identified in sample PT11370 (Table 4). The *bla* genes of samples PT11604 and PT11370 were both found in the middle of large contigs, being flanked by genes associated with mobile elements. For isolate PT12019, the *bla* gene was detected in a small contig with three more genes, which in the NCBI database, resulted in a hit with plasmid pLFS5 (96% query coverage and 99% identity).

The sequence of the *ftsI* gene (which codifies for PBP3) was examined in all genomes. In total, six *ftsI* alleles were detected: *ftsI* allele 2 was detected in serotype e isolates (*n* = 7), *ftsI* allele 6 was detected in all serotype f isolates (*n* = 22), *ftsI* allele 10 was detected in serotype b isolate (*n* = 1), *ftsI* allele 24 was detected in NTHi isolate (*n* = 1), *ftsI* allele 46 was detected in all serotype a isolates (*n* = 4) and *ftsI* allele 55 was detected in the remaining serotype e isolates (*n* = 7) (Table 4). The PBP3 mutations associated with β-lactams resistance were present in *ftsI* alleles: 2 (D350N, M377I, A502V and N526K, classified as BLNAR group IIb), 6 (D350N), 24 (D350N, A502T and N526K) and 55 (D350N). Isolates with *ftsI* alleles 2 and 24 showed resistance to some β-lactams (Table 4).

Mutations in the sequence of *folA* and *folP* genes, the molecular targets of trimethoprim-sulfamethoxazole, were also investigated. Changes in *folA* (F154S) and *folP* (P64E) were found for the three isolates (PT10981, PT11022 and PT11604) resistant to this antimicrobial agent (Table 4).

In all but two isolates, there was an agreement between the resistance phenotype and the genotype. The exceptions were: isolate PT10674 (serotype f), which was phenotypically resistant to cefepime, but no gene or mutation associated with β-lactam resistance could be detected on the transpeptidase region of PBP3, and isolate PT11703 (serotype f), which was resistant to ciprofloxacin (MIC = 0.12 mg/L), but no relevant mutations were found in the main target genes (*gyrA* and *parC*) that could justify the resistance phenotype.

### 3.7. Virulence Associated Genes

**Figure 1:**
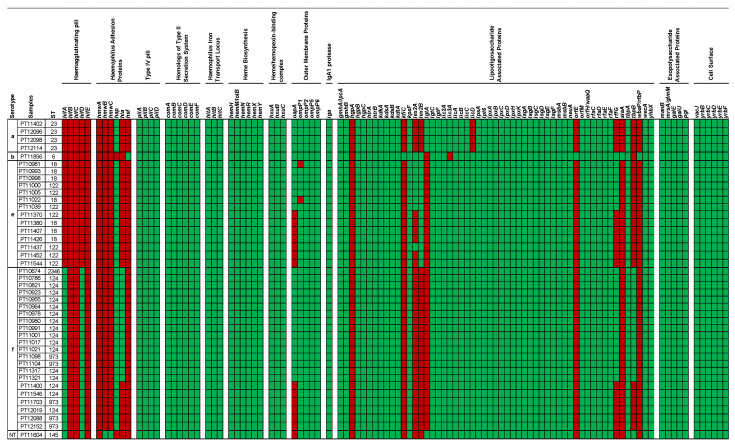
Detection of presence/absence of 105 virulence genes of *H. influenzae* that are described in [53,54]. Each line corresponds to a single isolate. The thicker borders delimit different serotypes. The first, second and third columns correspond to serotype, sample number and sequence type, respectively. Each other column corresponds to a single gene. Green squares show that the gene was detected. Red squares show that the gene was not detected.

To further characterize the samples, we searched for the presence of 105 genes previously associated with virulence in *H. influenzae* [53,54]. Only five genes were absent in all strains (*hifB*, *hifC* and *hifE*, all of which are associated with haemagglutinating pili; *hmwA*, which encodes for a *Haemophilus* adhesion protein; *hgpA,* which encodes for a hemoglobin binding protein). All other genes were represented in at least one strain, and most (77.1%, 81/105) of them were present in all strains (Figure 1).

There was a good concordance between the serotype and the presence of specific virulence genes. Still, the number of virulence genes did not vary much between serotypes (range: 85 to 91). The serotype b strain had the highest number of virulence genes (91 out of 105) (Figure 1).

### 3.8. Characterization of Cap Loci in H. influenzae and H. parainfluenzae

**Figure 2:**
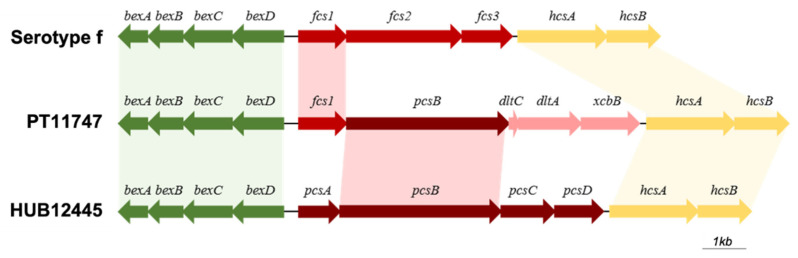
Schematic representation of serotype f and f-like cap loci. The first *cap* locus corresponds to the one that was obtained for PT11400 isolate (Hif), and it has the typical organization that is found for serotype f; the second locus represents the genetic organization of the *cap* locus of isolate PT11747 (*H. parainfluenzae*); the third locus shows the organization of the *cap* locus that was described for *H. parainfluenzae* HIU12445 [56]. Green arrows represent the genes from region I (*bex* genes cluster); red arrows represent region II (serotype specific genes); yellow arrows represent region III (*hcs* genes cluster). The orientation of the arrows indicates the direction of transcription. Shadows indicate homology between genes.

We investigated the presence and composition of the *cap* locus in the genomes of all sequenced strains. All *H. influenzae* strains previously identified by PCR as encapsulated, had a complete *cap* locus corresponding to the serotype determined by PCR. In addition, the *H. parainfluenzae* isolate (PT11747) had an incomplete *cap* locus: regions I and III were complete, but region II was incomplete when compared with the *cap* loci from *H. influenzae* (Figure 2). Region II codes for serotype specific genes in *H. influenzae*; in this case, however, one (*fcsI*) of the three specific genes of serotype f was present. Thus, we classified this sample as f-like. In addition, by using the hicap software, genome annotation, and blastp analysis, we could identify four putative additional genes in this region. Following the order of transcription, the second gene codes for a putative glycosyltransferase with homology (99.0% query coverage and 84.5% identity) with *pcsB* previously described in *H. parainfluenzae* strain HUB12445 [56]. The third and fourth genes were annotated as *dltC* and *dltA*, respectively, and their products were compared, by blastp in the NCBI database. The closest homologues were found in *Haemophilus haemolyticus* and these corresponded to an acyl carrier protein (100.0% query coverage and 98.7% identity) and an AMP-binding protein (100.0% query coverage and 97.0% identity), respectively. The fifth gene, initially annotated as a hypothetical protein, was identified, by blastp, as a gene coding for a capsular polysaccharide biosynthesis protein (*xcbB*) in *H. haemolyticus* (100.0% query coverage and 95.7% identity).

## 4. Discussion

Introduction of Hib vaccine has changed the epidemiology of *H. influenzae* infection and carriage, especially of serotype b [57,58]. As there are no recent data on *H. influenzae* carriage in Portugal, we aimed to characterize samples obtained from children attending day-care centers in the Lisbon region and investigate possible parallelisms with the disease epidemiology.

The prevalence of *H. influenzae* in carriers has been found to vary across different studies [5]. For example, studies from the Netherlands, Spain, France and Brazil reported carriage rates of 37%, 42%, 41% and 72%, respectively [38,39,40,59]. Lower rates were described more recently in two studies: 27% in 2015 in south of Spain [37], and 28% in 2016 in Indonesia [60].

We observed a high prevalence of nasopharyngeal colonization (84.1%), which is in line with our previous studies where carriage rates among day-care attendees varied between 71.5% and 87%, depending on the study [34,35]. In day-care centers, cross-transmission can be extremely high, which in turn, is reflected in the carriage rates observed [34].

Differences between carriage rates across different studies should be compared with caution, as factors such as geographic region, sampling season, individual and social factors and sampling method can influence the result obtained [5,37,58,61,62,63].

In our study, albeit carriage was uniformly high at all ages, it tended to peak during the second year of life and decline afterwards, thereby mimicking a general trend observed for this and other pathobionts [33,35,37,59,64,65,66,67].

Among all isolates, NTHi dominated with only 3.6% of the isolates being encapsulated. The latter belonged to serotypes f (1.8%), e (1.1%), a (0.3%) and b (0.1%). Serotypes e and f isolates were most frequent in 2016 (contributing to 5.9% and 2.9% of all isolates, respectively), serotype a was detected in 2018 and 2019, and a single isolate of serotype b was detected in 2019. The same serotypes have been found among isolates causing invasive disease in Portugal during the same study years [27]. Of note, in our previous study, which was carried out in the pre-vaccine period, the rate of serotype b carriage among the day-care center attendees was significantly higher (3.8%), thereby suggesting that the Hib vaccination program nearly eliminated carriage of this serotype [35]. Carriage of serotype a isolates, which may be as virulent as serotype b, is a matter of concern since this serotype has been increasingly described in invasive infections [27,68].

WGS was carried out for the encapsulated strains and for one NTHi strain that had a BLPACR phenotype (PT11604, with MIC = 4 mg/L to amoxicillin-clavulanic acid). MLST analysis showed that there was clonality within the serotypes, which is in agreement with previous studies [27,32,69,70,71]. In particular, serotype a isolates were of ST23, the serotype b isolate was of ST6, serotype e isolates were of ST18 or ST122 (single locus variants—SLVs of each other), and serotype f isolates were of ST124, ST973 or ST2346 (also SLVs of each other). The same lineages were found among isolates causing invasive disease in Portugal, during the same years [27]. Still, ST122, ST973 and ST2346 had not been found before. The only NTHi isolate analyzed by MLST was of ST145, a ST previously found in a single NTHi isolated in Portugal from CSF in 2013 [72]. In the PubMLST Database, isolates with this ST are all associated with invasive disease (https://pubmlst.org/hinfluenzae/database, last accessed on 1 August 2022). The association of ST145 to invasive disease suggests that this ST might have a higher invasive potential [67].

Regarding virulence genes, we detected an average of 86 virulence genes in each strain (ranging from 85 to 91) [53,54]. The isolate from serotype b is the one that presented a higher number of virulence genes. We observed that the presence/absence of virulence genes within each serotype was mostly constant, differing essentially between serotypes. Of interest, our colonization isolates tended to lack some virulence determinants when compared to strains of the same serotype isolated in Portugal from invasive disease specimens [72]. Specifically, among colonization isolates: (i) serotype a strains lacked *hsf*, *licD* and *rffG*; (ii) serotype b lacked *hap* and *lec3A*; (iii) serotype e strains lacked *hifD*, *oapA* and *rffG*; and (iv) serotype f strains lacked *hifB*, *hifC*, *hifE*, *oapA*, *lex2A*, *lex2B* and *rffG*. On the other hand, serotype e colonization isolates had *lex2B* and one strain contained *lex2A*, both absent from infection strains [72]. The abovementioned genes code for hemagglutinating pili (*hifB*, *hifC*, *hifD* and *hifE*), adhesion proteins (*hsf* and *hap*), OMPs (*oapA*) and lipooligoligosaccharide-associated proteins (*licD*, *lex2A*, *lex2B*, *lec3A* and *rffG*) [53,54]. Overall, these results suggest that, although there are some variations in the presence/absence of virulence genes, there are no major differences between the colonization and invasive strains.

An interesting finding was the identification of one *H. parainfluenzae* strain that had an incomplete *cap* locus. Regions I and III were complete, with four *bex* genes and two *hcs* genes, respectively. However, region II contained only one gene that is specific to serotype f (*fcsI*), being thus classified as an f-like serotype. In addition to this gene, four additional genes in region II were identified: *pcsB*, a gene that was previously identified in four strains of *H. parainfluenzae* containing a *cap* locus of *H. influenzae* [56]; and *dltC*, *dltA* and *xcbA*, which are homologous to genes from *H. haemolyticus.* Interestingly, the *cap* locus described here is different from the one described for *H. parainfluenzae* by González-Diaz and colleagues [56], as homology was only observed for regions I, II and the *pcsB* gene. *H. parainfluenzae* have long been considered as nonencapsulated [56]. To our best knowledge, this is the first encapsulated *H. parainfluenzae* identified in Portugal.

In our study, we investigated antimicrobial resistance phenotypes and genotypes. In general, concordance between both approaches was observed. Discrepancies were noted in two instances: one isolate was phenotypically resistant to ciprofloxacin (MIC = 0.12 mg/L), although no mutations that could justify this phenotype were found in the *gyrA* and *parC* genes [73,74,75]; another isolate was classified as being phenotypically resistant to cefepime (MIC = 0.5 mg/L), but no mutations associated with β-lactam resistance were found on the transpeptidase region of the PBP3. The discrepancies between the resistance phenotype and genotype could be due to differences in the MIC value, which can have one dilution difference which is associated with the methodology and/or to the reader. In both isolates, if MICs were one dilution lower (i.e., 0.06 and 0.25 mg/L for ciprofloxacin and cefepime, respectively), they would have been classified as susceptible.

On average, 7.5% of the isolates were β-lactamase producers, a proportion lower (13.5%) than the one observed among invasive disease isolates in Portugal in the period of 2011–2018 [27]. In addition, in our study, a decreasing trend was observed from 9.3% in 2015, 9.5% in 2016 and 8.5% in 2018, to 4.0% in 2019 (*p* = 0.001). Our previous studies, among children attending day-care centers in the pre-vaccine period, also showed a decline in the prevalence of β-lactamase-producing *H. influenzae* colonizing strains from 20.0% in 1996 to 11.6% in 1998–1999 [34,35]. As expected, all β-lactamase-producing isolates in our study were resistant to ampicillin with an MIC ≥ 4 mg/L. Of note, one β-lactamase producer isolate was phenotypically characterized as BLPACR (PT11604) with an MIC = 4 mg/L to amoxicillin-clavulanic acid.

Five of the whole-genome sequenced isolates were β-lactamase producers and genes encoding for β-lactamases were detected in these isolates: four had the *bla*_TEM-1_ gene; the other (PT11370, serotype e) had the *bla*_TEM-82_ gene. TEM-1 is the main type of β-lactamases in *H. influenzae* [46]. TEM-82 β-lactamases have been described in *Escherichia coli* as a variant of TEM-1 due to the substitution of two amino acids (M69V and R275Q) resulting in an Inhibitor Resistant TEM (IRT) [76]. To our best knowledge, *bla*_TEM-82_ has not been described before in *H. influenzae*. Isolates with TEM-82 remain susceptible to narrow- and extended-spectrum cephalosporins; however, they are resistant to ampicillin-sulbactam and are intermediately resistant or resistant to amoxicillin-clavulanate [77]. The phenotype resistance pattern of isolate PT11370 was resistance to ampicillin and intermediate resistance to cefuroxime. The MIC to amoxicillin-clavulanate was 2 mg/L, a value higher than the one observed for most isolates, although not resistant according to EUCAST breakpoints. This phenotype was previously observed when IRT β-lactamases were cloned in *H. influenzae* TEM-1 producer strains [78]. PT11370 was susceptible to all other antibiotics tested. Genotypically, it was classified as BLPACR and had mutations in *ftsI* associated with decreased affinity to β-lactams.

In addition, when analyzing non-enzymatic resistance mechanisms to ampicillin, we observed that within each ST, the *ftsI* sequence (coding for PBP3) was the same. Looking at the transpeptidase region of PBP3, only samples belonging to ST23 (serotype a) and ST6 (serotype b) showed no changes in amino acids considered relevant for resistance. Six isolates of serotype e were of gBLNAR group IIB (*ftsI* allele 2 resulting in PBP3 mutations D350N, M377I, A502V, and N526K). Two isolates, one of serotype e and the other a NTHi, were gBLPACR (PBP3 mutation in D350N M377I A502V N526K and D350N A502T N526K, respectively). Mutation N526K has been described as having an essential role in β-lactam non-susceptibility [45]; in agreement, isolates with this mutation showed this phenotype. Mutation M377I was previously associated with higher resistance to β-lactams antibiotics, namely to cefotaxime [79], but this was not observed in these two isolates.

Over one-third (34.4%) of the β-lactamase producer isolates were resistant to trimethoprim-sulfamethoxazole and mutations associated with this resistance phenotype were detected in *folA* and *folP* of three isolates analyzed by WGS [80]. High rates of resistance to trimethoprim-sulfamethoxazole were observed in previous studies [27,81,82].

Multidrug resistance was observed in three isolates only, all NTHi. Other studies have documented that with the decline of serotype b isolates, a parallel decline in resistance, especially multidrug resistance occurred [19,31,32].

To our best knowledge this study is the first colonization survey of *H. influenzae* carriage in children attending day-care centers in Portugal since the introduction of Hib vaccine in the NIP. The results obtained provided important insights with a focus on the estimation of the prevalence and genomic and phenotypic characteristics of encapsulated isolates. This study, however, has some limitations: (i) a single region of the country was surveilled, albeit including various day-care centers; (ii) antimicrobial susceptibility was only performed for β-lactamase producer and encapsulated isolates; and (iii) whole genome sequencing was only carried out for encapsulated isolates.

In conclusion, our results show that, after several years of introduction of Hib vaccine, circulation of Hib isolates is residual (<0.1%). The proportion of *H. influenzae* carriers among children attending day-care remains very high. Antimicrobial resistance has not increased among carried isolates. The few encapsulated strains in circulation are genotypically similar to the ones causing disease and thus surveillance of *H. influenzae* among carriers should be maintained.

## Figures and Tables

**Table 1:** Characteristics of population studied.

	Total		Year, *n* (%)							
Characteristic			2015		2016		2018		2019	
**Participants**	1524		315		318		427		464	
**Age group (months) ^a^**										
0–12	122	(8.0)	21	(6.7)	30	(9.6)	28	(6.6)	43	(9.4)
13–24	252	(16.6)	47	(14.9)	49	(15.6)	86	(20.2)	70	(15.2)
25–36	287	(19.0)	55	(17.5)	59	(18.8)	80	(18.8)	93	(20.2)
37–48	362	(23.9)	88	(27.9)	64	(20.4)	98	(23.1)	112	(24.3)
49–60	272	(18.0)	60	(19.0)	69	(29)	67	(15.8)	76	(16.5)
≥61	219	(14.5)	44	(14.0)	43	(13.7)	66	(15.5)	66	(14.4)
**Gender**										
Male	817	(53.6)	163	(51.8)	171	(53.8)	234	(54.8)	249	(53.7)
**Antimicrobial use**										
At sampling ^b^	63	(4.3)	14	(4.5)	11	(3.7)	21	(5.0)	17	(3.8)
In previous month ^c^	246	(17.6)	45	(15.6)	48	(17.8)	73	(18.1)	80	(18.5)
In previous six months ^d^	503	(36.1)	94	(33.0)	90	(31.4)	167	(42.8)	152	(35.3)

^a^ Data missing for 4 children in 2016, 2 children in 2018 and 4 children in 2019. ^b^ Data missing for 5 children in 2015, 19 children in 2016, 8 children in 2018 and 22 children in 2019. ^c^ Data missing for 26 children in 2015, 48 children in 2016, 23 children in 2018 and 31 children in 2019. ^d^ Data missing for 30 children in 2015, 31 children in 2016, 37 children in 2018 and 33 children in 2019.

**Table 2:** Colonization rates and capsular types.

*H. influenzae* Carriage	Total	Year, % (*n*/Total)			
		2015	2016	2018	2019
**Global**	84.1 (1282/1524)	88.6 (279/315)	75.2 (239/318)	90.6 (387/427)	81.3 (377/464)
**by age group (months) ^a^**					
0–12	83.6 (102/122)	81.0 (17/21)	73.3 (22/30)	92.9 (26/28)	86.0 (37/43)
13–24	92.1 (232/252)	91.5 (43/47)	89.8 (44/49)	95.3 (82/86)	90.0 (63/70)
25–36	86.4 (248/287)	92.7 (51/59)	81.4 (48/59)	91.3 (73/80)	81.7 (76/93)
37–48	84.3 (305/362)	92.0 (81/88)	73.4 (47/64)	89.8 (88/98)	79.5 (89/112)
49–60	82.4 (224/272)	86.7 (52/60)	76.8 (53/69)	86.6 (58/67)	80.3 (61/76)
≥61	75.3 (165/219)	79.5 (35/44)	51.2 (22/43)	90.9 (60/66)	72.7 (48/66)
**by serotype**					
serotype a	0.3 (4/1282)	0.0 (0/279)	0.0 (0/239)	0.3 (1/387)	0.8 (3/377)
serotype b	0.1(1/1282)	0.0 (0/279)	0.0 (0/239)	0.0 (0/387)	0.3 (1/377)
serotype e	1.1(14/1282)	0.0 (0/279)	2.9 (7/239)	1.8 (7/387)	0.0 (0/377)
serotype f	1.8 (23/1282)	0.7 (2/279)	5.9 (14/239)	0.8 (3/387)	1.0 (4/377)
nonencapsulated	96.7 (1240/1282)	99.3 (277/279)	91.2 (218/239)	97.1 (376/387)	97.9 (369/377)

^a^ Data missing for 3 children in 2016 and 3 children in 2019.

**Table 3:** Antimicrobial susceptibility of β-lactamase producers and encapsulated isolates.

Antimicrobial Agent	β-Lactamase Producers (*n* = 96) ^a^						Capsulated (*n* = 42) ^a^					
	S (%)		I (%)		R (%)		S (%)		I (%)		R (%)	
Ampicillin	0	(0.0)	0	(0.0)	96	(100.0)	37	(88.1)	0	(0.0)	5	(11.9)
Amoxicillin-clavulanic acid	95	(99.0)	0	(0.0)	1	(1.0)	42	(100.0)	0	(0.0)	0	(0.0)
Cefepime	93	(96.9)	0	(0.0)	3	(3.1)	41	(97.6)	0	(0.0)	1	(2.4)
Cefotaxime	96	(100.0)	0	(0.0)	0	(0.0)	42	(100.0)	0	(0.0)	0	(0.0)
Cefuroxime	0	(0.0)	87	(90.6)	9	(9.4)	0	(0.0)	40	(95.2)	2	(4.8)
Ciprofloxacin	95	(99.0)	0	(0.0)	1	(1.0)	42	(100.0)	0	(0.0)	0	(0.0)
Chloramphenicol	93	(96.9)	0	(0.0)	3	(3.1)	42	(100.0)	0	(0.0)	0	(0.0)
Meropenem	96	(100.0)	0	(0.0)	0	(0.0)	42	(100.0)	0	(0.0)	0	(0.0)
Tetracycline	94	(97.9)	0	(0.0)	2	(2.1)	42	(100.0)	0	(0.0)	0	(0.0)
Trimethoprim-sulfamethoxazole	63	(65.6)	0	(0.0)	33	(34.4)	40	(95.2)	0	(0.0)	2	(4.8)
Rifampicin	96	(100.0)	0	(0.0)	0	(0.0)	42	(100.0)	0	(0.0)	0	(0.0)

^a^ Five isolates are both β-lactamase producers and encapsulated *Haemophilus influenzae* and are included in both groups. S, susceptible; I, intermediate resistant; R, resistant.

**Table 4:** *H. influenzae* isolates analyzed by whole-genome sequencing.

Strain Reference	Sampling Year	Genome Size (Mbp)	Genome Coverage	Number of Contigs	Serotype	Multilocus Sequence Typing								Phenotype Resistance Pattern	Antimicrobial Resistance Determinants					
						*adk*	*atpG*	*frdB*	*fucK*	*mdh*	*pgi*	*recA*	ST		Resistance Genotype	*bla* Gene	*ftsI* Allele	PBP3 Mutations ^a^	*folA* Mutations ^a^	folP Mutations ^a^
PT12096	2019	1.76	352	32	**a**	13	16	5	2	3	11	7	23	-	-	nd	46	-		
PT12098	2019	1.76	323	30	**a**	13	16	5	2	3	11	7	23	-	-	nd	46	-		
PT12114	2019	1.76	292	33	**a**	13	16	5	2	3	11	7	23	-	-	nd	46	-		
PT11402	2018	1.76	27	46	**a**	13	16	5	2	3	11	7	23	-	-	nd	46	-		
PT11856	2019	1.87	217	46	**b**	10	14	4	5	4	7	8	6	-	-	nd	10	-		
PT10981	2016	1.85	243	33	**e**	18	6	3	7	10	28	12	18	Amp, Cfx(I),SXT, BLP	BLP	*bla* TEM-1	55	D350N	F154S	P64E
PT10993	2016	1.84	287	37	**e**	18	6	3	7	10	28	12	18	-	-	nd	55	D350N		
PT10998	2016	1.84	291	37	**e**	18	6	3	7	10	28	12	18	-	-	nd	55	D350N		
PT11000	2016	1.84	307	31	**e**	18	6	3	18	10	28	12	122	Cfx (I)	BLNAR IIb	nd	2	D350N M377I A502V N526K		
PT11005	2016	1.84	308	29	**e**	18	6	3	18	10	28	12	122	Cfx	BLNAR IIb	nd	2	D350N M377I A502V N526K		
PT11022	2016	1.84	101	36	**e**	18	6	3	7	10	28	12	18	Amp, Cfx(I),SXT, BLP	BLP	*bla* TEM-1	55	D350N	F154S	P64E
PT11039	2016	1.84	190	33	**e**	18	6	3	18	10	28	12	122	Cfx	BLNAR IIb	nd	2	D350N M377I A502V N526K		
PT11370	2018	1.90	52	41	**e**	18	6	3	18	10	28	12	122	Amp, Cfx(I), BLP	BLPACR	*bla* TEM-82	2	D350N M377I A502V N526K		
PT11380	2018	1.84	43	32	**e**	18	6	3	7	10	28	12	18	-	-	nd	55	D350N		
PT11407	2018	1.84	27	40	**e**	18	6	3	7	10	28	12	18	-	-	nd	55	D350N		
PT11426	2018	1.84	32	31	**e**	18	6	3	7	10	28	12	18	-	-	nd	55	D350N		
PT11437	2018	1.84	35	35	**e**	18	6	3	18	10	28	12	122	Cfx (I)	BLNAR IIb	nd	2	D350N M377I A502V N526K		
PT11452	2018	1.84	25	43	**e**	18	6	3	18	10	28	12	122	Cfx (I)	BLNAR IIb	nd	2	D350N M377I A502V N526K		
PT11544	2018	1.84	33	39	**e**	18	6	3	18	10	28	12	122	Cfx (I)	BLNAR IIb	nd	2	D350N M377I A502V N526K		
PT10674	2015	1.81	262	21	**f**	22	19	11	11	86	19	15	2346	Cfx (I), Cpe	-	nd	6	D350N		
PT10786	2015	1.81	222	23	**f**	22	19	11	11	22	19	15	124	-	-	nd	6	D350N		
PT10821	2016	1.81	290	20	**f**	22	19	11	11	22	19	15	124	-	-	nd	6	D350N		
PT10923	2016	1.81	195	21	**f**	22	19	11	11	22	19	15	124	-	-	nd	6	D350N		
PT10955	2016	1.81	266	22	**f**	22	19	11	11	22	19	15	124	-	-	nd	6	D350N		
PT10964	2016	1.81	319	25	**f**	22	19	11	11	22	19	15	124	-	-	nd	6	D350N		
PT10978	2016	1.81	335	22	**f**	22	19	11	11	22	19	15	124	-	-	nd	6	D350N		
PT10980	2016	1.81	236	24	**f**	22	19	11	11	22	19	15	124	-	-	nd	6	D350N		
PT10991	2016	1.81	238	23	**f**	22	19	11	11	22	19	15	124	-	-	nd	6	D350N		
PT11001	2016	1.81	324	25	**f**	22	19	11	11	22	19	15	124	-	-	nd	6	D350N		
PT11017	2016	1.81	258	22	**f**	22	19	11	11	22	19	15	124	-	-	nd	6	D350N		
PT11021	2016	1.81	91	21	**f**	22	19	11	11	22	19	15	124	-	-	nd	6	D350N		
PT11098	2016	1.81	78	24	**f**	22	19	140	11	22	19	15	973	-	-	nd	6	D350N		
PT11104	2016	1.81	75	24	**f**	22	19	140	11	22	19	15	973	-	-	nd	6	D350N		
PT11317	2016	1.81	73	24	**f**	22	19	11	11	22	19	15	124	-	-	nd	6	D350N		
PT11321	2016	1.81	66	21	**f**	22	19	11	11	22	19	15	124	-	-	nd	6	D350N		
PT11400	2018	1.81	53	21	**f**	22	19	11	11	22	19	15	124	-	-	nd	6	D350N		
PT11546	2018	1.81	37	29	**f**	22	19	11	11	22	19	15	124	-	-	nd	6	D350N		
PT11703	2018	1.81	156	20	**f**	22	19	140	11	22	19	15	973	Cip	-	nd	6	D350N		
PT12019	2019	1.81	308	23	**f**	22	19	11	11	22	19	15	124	Amp, Cfx(I), BLP	BLP	*bla* TEM-1	6	D350N		
PT12088	2019	1.81	286	22	**f**	22	19	140	11	22	19	15	973	-	-	nd	6	D350N		
PT12152	2019	1.81	298	21	**f**	22	19	140	11	22	19	15	973	-	-	nd	6	D350N		
PT11604	2018	1.84	84	101	**NT**	1	8	1	14	22	14	13	145	Amp, Aug, Cfx, Cpe, SXT, BLP	BLPACR	*bla* TEM-1	24	D350N A502T N526K	F154S	P64E

^a^ The amino acids used for comparison are those of reference strain Rd KW20 (accession code: NC_000907). Amino acid substitutions are written as follows: D350N, meaning that a D (aspartic acid) at position 350 of the gene of the reference strain has been replaced by N (asparagine). D, aspartic acid; N, asparagine; M, methionine; I, isoleucine; A, alanine; V, valine; K, lysine; T, threonine; F, phenylalanine; S, serine P, proline; E, glutamic acid, Amp, ampicillin; Aug, Amoxicillin-clavulanic acid; Cfx, cefuroxime; Cip, Ciprofloxacin; Cpe, Cefepime; SXT, trimethoprim–sulfamethoxazole; I, intermediate; nd, not detected; BLNAS, β-lactamase-negative ampicillin-susceptible; BLNAR, β-lactamase-negative ampicillin-resistant; BLP, β-lactamase-positive; BLPACR, β-lactamase-positive amoxicillin/clavulanic acid-resistant.

## Data Availability

Aggregated data generated or analyzed during this study are included in this article. WGS data are available on BioProject accession number PRJNA824278. Additional information on the datasets is available from RSL upon reasonable request.
